# 循环肿瘤细胞中不同表型细胞*FGFR1*基因表达程度与非小细胞肺癌临床病理特点相关性研究

**DOI:** 10.3779/j.issn.1009-3419.2018.05.03

**Published:** 2018-05-20

**Authors:** 磊 刘, 诚 黄, 力 李, 乃新 梁, 单青 李

**Affiliations:** 1 100730 北京，中国医学科学院北京协和医学院，北京协和医院胸外科 Department of Thoracic Surgery, Peking Union Medical College Hospital, Chinese Academy of Medical Sciences & Peking Union Medical College, Beijing 100730, China; 2 100730 北京，中国医学科学院北京协和医院胸外科 Department of Thoracic Surgery, Peking Union Medical College Hospital, Chinese Academy of Medical Sciences, Beijing 100730, China

**Keywords:** 肺肿瘤, 循环肿瘤细胞, 成纤维细胞生长因子受体1, 吸烟史, 转移, Lung neoplasms, Circulating tumor cell, Fibroblast growth factor receptor 1, Smoking history, Metastasis

## Abstract

**背景与目的:**

目前检测非小细胞肺癌（non-small cell lung cancer, NSCLC）术后患者复发转移的方法均具有一定的滞后性及片面性。本研究总结分析了30例NSCLC患者外周血循环肿瘤细胞（circulating tumor cell, CTC）及成纤维细胞生长因子受体1（fibroblast growth factor receptor 1, FGFR1）表达情况与临床病理之间的关系，以期能够为肿瘤复发转移的检测提供新思路。

**方法:**

分析北京协和医院胸外科2016年11月-2017年6月30例NSCLC患者临床资料及CTC检测数据并进行相关性分析。

**结果:**

相关性数据分析可得，外周血CTC细胞阳性率与吸烟史相关（*P*=0.016），病理类型与CTC阳性率及FGFR1表达情况之间无明显关联（*P*=0.202, *P*=0.806），不同类型CTC细胞FGFR1表达情况并无明显差异（*P*=0.094）。

**结论:**

CTC阳性率与NSCLC患者吸烟史相关，不同病理类型NSCLC中CTC分类及FGFR1表达情况无明显差异，不同类型CTC之间FGFR1表达情况无明显差异。我们期待着更大样本量及纳入随访数据后可得出与CTC及*FGFR1*基因表达相关的更多具有临床应用意义的结论。

## Abstract (en)

**Background and objective:**

The methods of detection for recurrence and metastasis in patients with non-small cell lung cancer (NSCLC) have hysteresis and one-sidedness. This study summarizes the relationship between the circulating tumor cell (CTC) in peripheral blood, expression of fibroblast growth factor receptor 1 (FGFR1) and clinic pathological features in 30 patients with NSCLC so as to provide new ideas for the detection of tumor recurrence and metastasis.

**Methods:**

To analyze the clinical data and CTC detection data of 30 cases of NSCLC in Department of Thoracic Surgery, Peking Union Medical College Hospital from November 2016 to June 2017.

**Results:**

Data analysis showed that the positive rate of CTC in peripheral blood was remarkably correlated with the smoking history (*P*=0.016). There was no significant correlation among the pathological type and CTC positive rate and the expression of FGFR1 (*P*=0.202, *P*=0.806). There was no significant difference in the expression of FGFR1 in different type CTC cells (*P*=0.094).

**Conclusion:**

The positive rate of CTC was significantly correlated with the smoking history of patients with NSCLC. There was no significant difference in CTC classification and FGFR1 expression in different pathological types of NSCLC. There was no significant difference in the expression of FGFR1 between different types of CTCs. We look forward to a larger sample size and inclusion of follow-up data to arrive at more clinically relevant conclusions about CTC and *FGFR1* gene expression.

肺癌已成为恶性肿瘤相关死亡主要原因之一^[1]^，其中非小细胞肺癌（non-small cell lung cancer, NSCLC）约占肺癌的85%。目前，对于早期NSCLC的主要治疗方式以手术为主，但是仍有30%-70%的术后患者出现复发或转移的情况。Ia期、Ib期、Ⅱa期、Ⅱb期及Ⅲa期NSCLC患者的5年生存率分别为50%、43%、36%、25%及19%^[2, 3]^。肿瘤复发和转移成为NSCLC患者死亡的直接原因。目前针对NSCLC肿瘤转移及复发的筛查方法均有一定的滞后性和片面性。因此对于NSCLC临床病理特征全面准确的评估并根据此制定有针对性的个体化肿瘤复发监测方案就变得尤为重要。

随着近几年对肿瘤基因的研究，发现成纤维细胞生长因子受体在肿瘤发生发展的过程中起着重要的作用。既往研究已发现成纤维细胞生长因子受体1（fibroblast growth factor receptor 1, FGFR1）在胃癌、结直肠癌及骨肉瘤中均有扩增表达^[3-5]^。最近发现FGFR1在NSCLC发生过程中也具有一定作用^[6]^。进一步的研究发现FGFR1与NSCLC鳞癌亚型、男性及吸烟史等临床特点相关^[7, 8]^。

在生物技术发展的推动下，血液循环肿瘤细胞（circulating tumor cells, CTC）因其实时、无创、灵敏度高等特点在肿瘤复发转移监测中得到逐步广泛的应用。Hofman等^[2]^的一项大样本前瞻性研究显示，术前CTCs值可用来预测接受根治性手术切除的Ⅰ期-Ⅱ期NSCLC患者的无疾病生存期（disease-free survival, DFS）与总体生存时间（overall survival, OS），对于术前CTCs > 5个的患者其DFS要更短一些，有更高的复发风险。

本研究纳入了30例NSCLC患者，在术前1周内留取患者外周血，检测其外周血中CTC数量，同时对CTC进行分型，并在此基础上检测不同类型CTC中*FGFR1*基因表达的程度，分析不同类型CTC中FGFR1表达与NSCLC临床病理特点之间的关系。

## 1 资料与方法

收集北京协和医院胸外科2016年11月-2017年6月30例组织学或细胞学确认为NSCLC患者外周血5 mL，并对其进行检测。

### 1.1 患者筛选

#### 1.1.1 入选标准

① 年龄≥18岁，性别不限；②组织学或细胞学确认为NSCLC（腺癌、鳞状细胞癌、大细胞癌等）患者，共留取30例患者标本；③初诊后未经过任何治疗（包括术前化疗、手术等）；④签署知情同意书。

#### 1.1.2 排除标准

① 术后组织学或细胞学证实为小细胞肺癌、良性肿瘤等非NSCLC患者；②术前采集外周血标本数量不足（< 5 mL）；③外周血标本不符合检测要求，发生溶血、凝血等，影响最终检测结果；④已入组患者要求退出试验。

### 1.2 观察指标

① CTC阳性率，其中包括不同表型（上皮型、混合型及间质型）各自阳性率；②FGFR1表达的程度，包括无表达、低表达、中表达及高表达；③患者一般情况，包括性别、年龄、吸烟史、肿瘤病史、家族史等；④NSCLC病理检验情况，包括肿瘤类型、原发实体肿瘤大小、淋巴结转移情况等。

### 1.3 CTC分离分型及FGFR1表达检测

#### 1.3.1 CanPatrol技术介绍及CTC检测原理

该技术将纳米膜过滤技术和多重RNA原位分析技术进行结合。首先裂解外周血中的红细胞，后利用CTCs与白细胞的大小差异，采用经自主优化生产的8 μm孔径纳米膜进行过滤及富集。然后通过多重mRNA原位分析（multiple mRNA *in situ* analysis, MRIA）方法对富集的CTCs进行特异性基因核酸定位，达到对CTCs进行分型和鉴定的目的，所用到的核酸及探针序列见表 1。RNA探针与目的基因杂交后，通过荧光信号放大体系，提高检测灵敏度至单拷贝mRNA。本技术可同时标记多种针对CTCs的RNA探针，分别对表达上皮型基因的CTCs和表达间质型的CTCs进行不同荧光染料标记，进而对其进行鉴定和分型。

**1:** CTC分型鉴定所用探针序列 Probe sequence used in the identification of CTC classification

Gene	Sequences (5’→3’)
*EpCAM*	TGGTGCTCGTTGATGAGTCA AGCCAGCTTTGAGCAAATGA AAAGCCCATCATTGTTCTGG CTCTCATCGCAGTCAGGATC TCCTTGTCTGTTCTTCTGAC CTCAGAGCAGGTTATTTCAG
*Vimentin*	GAGCGAGAGTGGCAGAGGAC CTTTGTCGTTGGTTAGCTGG CATATTGCTGACGTACGTCA GAGCGCCCCTAAGTTTTTAA AAGATTGCAGGGTGTTTTCG GGCCAATAGTGTCTTGGTAG
*CK8*	CGTACCTTGTCTATGAAGGA ACTTGGTCTCCAGCATCTTG CCTAAGGTTGTTGATGTAGC CTGAGGAAGTTGATCTCGTC CAGATGTGTCCGAGATCTGG TGACCTCAGCAATGATGCTG
*CK18*	AGAAAGGACAGGACTCAGGC GAGTGGTGAAGCTCATGCTG TCAGGTCCTCGATGATCTTG CAATCTGCAGAACGATGCGG AAGTCATCAGCAGCAAGACG CTGCAGTCGTGTGATATTGG
*CK19*	CTGTAGGAAGTCATGGCGAG AAGTCATCTGCAGCCAGACG CTGTTCCGTCTCAAACTTGG TTCTTCTTCAGGTAGGCCAG CTCAGCGTACTGATTTCCTC GTGAACCAGGCTTCAGCATC
*Twist*	ACAATGACATCTAGGTCTCC CTGGTAGAGGAAGTCGATGT CAACTGTTCAGACTTCTATC CCTCTTGAGAATGCATGCAT TTTCAGTGGCTGATTGGCAC TTACCATGGGTCCTCAATAA
*CD45*	TCGCAATTCTTATGCGACTC TGTCATGGAGACAGTCATGT GTATTTCCAGCTTCAACTTC CCATCAATATAGCTGGCATT TTGTGCAGCAATGTATTTCC TACTTGAACCATCAGGCATC
CTC: circulating tumor cell.	

#### 1.3.2 样本采集

使用8号采血针和EDTA抗凝采血管采集外周血样本并进行预处理。

#### 1.3.3 CTCs富集

将预处理后的样本抽滤至过滤器中。

#### 1.3.4 基因探针杂交

使用特异性捕获探针与目标mRNA进行杂交。

#### 1.3.5 扩增探针杂交

使用扩增探针与捕获探针进行杂交，为杂交信号的放大做准备。

#### 1.3.6 信号标记探针杂交

使用标记有荧光基团的标记探针与扩增探针进行杂交，产生荧光信号。

#### 1.3.7 自动识别

利用自动识别系统阅读荧光信号，自动判断检测结果。

#### 1.3.8 多重RNA原位分析检测

透化剂孵育5 min，PBS洗涤三次。消化酶孵育60 min，PBS洗涤3次。加入探针工作液，置于（40±1）℃生化培养箱中，孵育3 h。洗涤液洗涤3次。加入预扩增工作液，置于（40±1）℃生化培养箱中，孵育30 min。洗涤液洗涤3次。加入扩增工作液，置于（40±1）℃生化培养箱中，孵育30 min。洗涤液洗涤3次。加入显色工作液，置于（40±1）℃生化培养箱中，孵育30 min。洗涤液洗涤3次。在样本上加抗淬灭剂（含DAPI）。样本放置5 min后直接进行结果观察。结果分析：采用多重RNA探针，分别针对多种CTCs特异性基因，通过不同颜色荧光信号，可进一步将CTCs分型。其中Ⅰ型CTCs显示为红色荧光信号点，Ⅲ型显示为绿色荧光信号点，同时表达Ⅰ型和Ⅲ型特异性基因的CTCs为Ⅱ型（同时显示红色荧光及绿色荧光信号点）。CTCs分型标准见表 2，镜下判读见图 1。

**2:** CTC分型标准 Standard of CTC classification

	Type	Red fluorescent signal point	Green fluorescent signal point	Bluefluorescent signal point	DAPI
CTCs	Ⅰ	+	-	-	+
	Ⅱ	+	+	-	+
	Ⅲ	-	+	-	+

**1:**
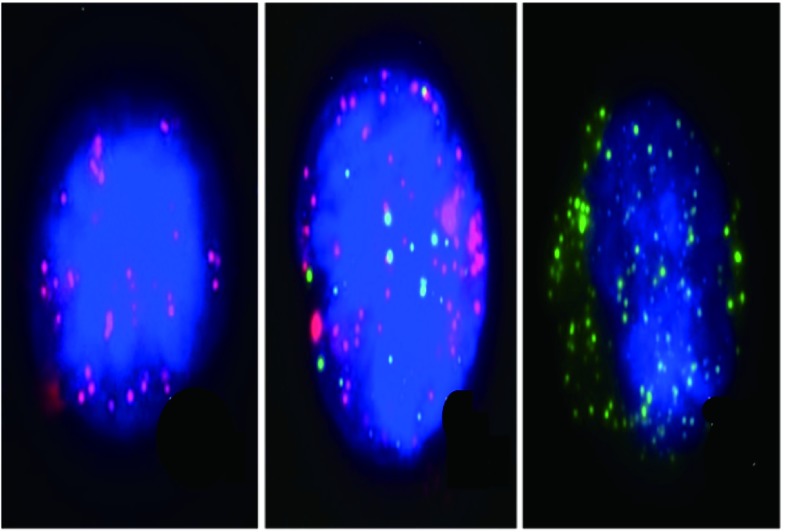
镜下判定三种不同类型的CTC（从左至右）：间质型、混合型、上皮型 Three different types of CTC under microscope (from left to right): mesenchymal type, mixed type, epithelial type

#### 1.3.9 *FGFR1*基因表达检测

前序步骤同上，在CTC富集的基础上，应用FGFR1捕获探针及扩增探针，利用荧光染料Alexa Flour 647进行标记，标记为紫色，捕获探针及扩增探针序列见表 3和表 4。基因表达程度判定标准为：信号点没有为无表达，1个-2个信号点为低表达，3个-9个信号点为中表达，≥10个信号点为高表达。

**3:** FGFR1捕获序列 FGFR1 capture sequence

Gene	Sequences (5’→3’)
*FGFR1*	AAGGTCCGTTATGCCACCTG GTGGTGCCCTCTGACAAGGG ATGTCGTGGAGCGGTCCCCT GGGTAGCAACGTGGAGTTCA GTACAGTGACCCGCAGCCGC GCCCAGACAACCTGCCTTAT AGAGATGGAGGTGCTTCACT GACGCAGGGGAGTATACGTG
FGFR1: fibroblast growth factor receptor 1.	

**4:** *FGFR1*扩增序列 *FGFR1* amplification sequence

	Function (copies)	Sequence (5′→3′)	Complement	Fluorescent dyes
bDNA probes for EpCAM and CK8/18/19	Capture probe tail (1)	CTACAAACAAACAATATT	Preamplifier leader (1)	Alexa Fluor 594
	Preamplifier repeat (5)	CGCAGCCTCAGCC	Amplifier leader (1)	
	Amplifier repeat (5)	CCCAGACCCTACC	Label probe (1)	
bDNA probes for vimentin and twist	Capture probe tail (1)	CTTCTCAATAACTAACAT	Preamplifier leader (1)	Alexa Fluor 488
	Preamplifier repeat (5)	GACGGTCGGCGTT	Amplifier leader (1)	
	Amplifier repeat (5)	GTCACCGCTCCAC	Label probe (1)	
bDNA probes for FGFR1	Capture probe tail (1)	CTTTATACCTTTCTTTCA	Preamplifier leader (1)	Alexa Fluor 647
	Preamplifier repeat (5)	GCGCGCTGTAGGG	Amplifier leader (1)	
	Amplifier repeat (5)	AGGCGAGGGGAGA	Label probe (1)	
bDNA probes for CD45	Capture probe tail (1)	GTAAAAAGAAAGGTATAA	Preamplifier leader (1)	Alexa Fluor 750
	Preamplifier repeat (5)	AATTATACATCTC	Amplifier leader (1)	
	Amplifier repeat (5)	GAAATGAATGAAT	Label probe (1)	

### 1.4 统计学方法

数据分析利用SPSS 24.0软件进行统计学分析。不同组数据之间的关系应用秩相关并采取*Spearman*秩相关系数进行分析。两组不同数据间分布差异性分析采用*Mann-Whitney U*检验方法，多组间数据分布差异性分析采用*Kruskal-Wallis H*检验。*P* < 0.05为有统计学差异。

## 2 结果

### 2.1 入组患者临床及病理特征

在入组的30例患者中，男性患者18例，女性患者12例。入组患者平均年龄64.5岁。入组患者中，腺癌患者15例，鳞癌患者15例。入组患者中肿瘤分期自Ⅰ期-Ⅳ期均有分布，其中Ⅰ期患者15例，Ⅱ期患者5例，Ⅲ期患者6例，Ⅳ期患者4例。入组患者中28例患者外周血中检测出CTC，CTC数量自1个-25个不等，平均为6.4个。本组患者中上皮型CTC共检出71个，平均2.36个，混合型CTC共检出97个，平均3.23个，间质型CTC共检出25个，平均0.83个，镜下分型情况见图 2。检测出的CTC中，*FGFR1*基因无表达CTC检测出151个，低表达CTC共检测出16个，中表达CTC共检测出12个，高表达CTC共检测出14个（图 2）。目前本组患者一般情况及检测结果见表 5及表 6。

**2:**
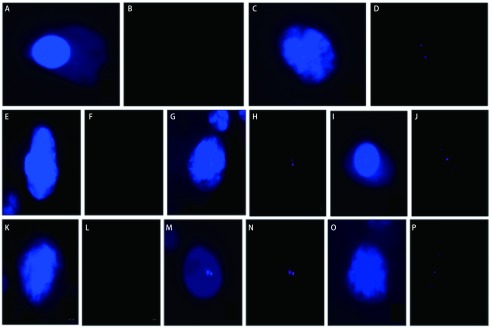
镜下判定不同分型CTC *FGFR1*基因表达情况。间质型CTC：A-B：无表达，C-D：低表达；混合型CTC：E-F：无表达，G-H：低表达，I-J：中表达；上皮型CTC：K-L：无表达，M-N：低表达，O-P：中表达。 The *FGFR1* gene expression of different types of CTC under microscope. Mesenchymal CTC: A-B: no expression, C-D: low expression; mixed CTC: E-F: no expression, G-H: low expression, I-J: middle expression; epithelial CTC: K-L: no expression, M-N: low expression, O-P: middle expression.

**5:** 入组患者临床特征 Clinical characteristics of patients included

Clinical and pathological features	Data
Total	30 (100.00%)
Gender	
Male	18 (60.00%)
Female	12 (40.00%)
Average age (yr)	64.5
Smoking history	
Yes	16 (53.33%)
No	14 (46.67%)
Pathological type	
Adenocarcinoma	15 (50.00%)
Squamous cell carcinoma	15 (50.00%)
T staging	
T1	15 (50.00%)
T2	9 (30.00%)
T3	1 (3.33%)
T4	5 (16.67%)
N staging	
N0	19 (63.33%)
N1	5 (16.67%)
N2	6 (20.00%)
M staging	
M0	26 (86.67%)
M1	4 (13.33%)
TNM staging	
Stage Ⅰ	15 (50%)
Stage Ⅱ	5 (16.67%)
Stage Ⅲ	6 (20.00%)
Stage Ⅳ	4 (13.33%)
TNM: tumor-node-metastasis.	

**6:** CTC及FGFR1表达情况检测结果 Results of detection for CTC and FGFR1 expression

No.	CTC	CTC type and FGFR1 expression (case)													
		Epithelial type					Mixed type					Mesenchymal type			
		N	L	M	H		N	L	M	H		N	L	M	H
1	21	0	0	0	0		0	0	6	13		0	0	2	0
2	6	0	0	0	0		1	0	0	0		3	2	0	0
3	9	3	0	0	0		4	0	0	0		2	0	0	0
4	25	14	0	0	0		10	0	0	0		1	0	0	0
5	6	0	0	0	0		1	0	0	0		3	2	0	0
6	1	1	0	0	0		0	0	0	0		0	0	0	0
7	1	0	0	0	0		0	0	0	0		0	0	0	0
8	5	0	0	0	0		4	0	1	0		0	0	0	0
9	4	1	0	1	0		0	0	1	0		1	0	0	0
10	2	0	0	0	0		2	0	0	0		0	0	0	0
11	14	4	0	0	0		10	0	0	0		0	0	0	0
12	3	0	0	0	0		2	1	0	0		0	0	0	0
13	5	3	0	0	0		2	0	0	0		0	0	0	0
14	0	0	0	0	0		0	0	0	0		0	0	0	0
15	5	0	0	0	0		4	0	0	1		0	0	0	0
16	7	7	0	0	0		0	0	0	0		0	0	0	0
17	5	2	0	0	0		3	0	0	0		0	0	0	0
18	13	5	2	0	0		4	1	0	0		1	0	0	0
19	0	0	0	0	0		0	0	0	0		0	0	0	0
20	3	0	0	0	0		3	0	0	0		0	0	0	0
21	3	0	0	0	0		3	0	0	0		0	0	0	0
22	11	5	1	0	0		3	0	0	0		1	1	0	0
23	3	0	0	0	0		2	0	1	0		0	0	0	0
24	11	9	0	0	0		1	1	0	0		0	0	0	0
25	3	0	0	0	0		0	0	0	0		1	2	0	0
26	7	1	0	0	0		1	2	0	0		3	0	0	0
27	8	8	0	0	0		0	0	0	0		0	0	0	0
28	2	0	0	0	0		2	0	0	0		0	0	0	0
29	1	0	0	0	0		0	1	0	0		0	0	0	0
30	10	4	0	0	0		6	0	0	0		0	0	0	0
N: no expression; L: low expression; M: middle expression; H: high expression.															

### 2.2 数据分析结果

整理上述数据，定义CTC阳性结果为外周血中CTC细胞数≥5个，CTC细胞中FGFR1无表达定义为无表达状态，低表达、中表达及高表达水平定位为表达状态，利用秩相关分方法，采用*Spearman*秩相关系数对所得的数据进行分析。根据目前数据分析可得，外周血CTC细胞是否阳性与吸烟史相关（*Spearman*系数=0.434，*P*=0.016）在秩和检验中，*Mann-Whitney U*检验病理类型与CTC阳性及*FGFR1*基因表达情况之间的关系，发现不同类型肺癌患者外周血CTC阳性率与*FGFR1*基因表达情况并无明显差异（*P*=0.202, *P*=0.806），*Kruskal-Wallis*检验发现不同类型CTC细胞FGFR1表达情况并无明显差异（*P*=0.094），相关数据见表 7。

**7:** 秩相关分析列联表 R×C table of rank correlation

Distribution of CTC positive rate in patients with different smoking history				
Smoking history	CTC detectionresults	Positive	Negative
Yes		10	6
No		3	11
Expression of FGFR1 in CTC of patients with different pathological types of NSCLC				
Pathological type	FGFR1 expression	Yes	No
Adenocarcinoma		7	8
Squamous carcinoma		6	9
Distribution of CTC positive rate in NSCLC patients with different pathological types				
Pathological type	CTC positive rate	Positive	Negative
Adenocarcinoma		8	7
Squamous carcinoma		8	7
FGFR1 expression in different types of CTC				
CTC type	FGFR1 expression	Yes	No
Epithelial type		3	27
Mixed type		10	20
Mesenchymal type		5	25

## 3 讨论

随着NSCLC发病率及死亡率的不断升高，如何能够快速准确的对其进行早期诊断并监测肿瘤复发转移越来越引起人们的关注。CTC在1869年由Thomas Ashworth首次发现。随着CTC检测技术的逐渐发展，发现CTC与肿瘤的分型、分期、预后以及转移均有着密切的关系。现临床最常使用的CTC检测技术是Cellsearch技术，是目前唯一被美国食品药品监督管理局批准用于临床CTC检测的方法，但是该方法在临床使用上的局限性较大：①该方法只能检测到带上皮细胞粘附分子（epithelial cell adhesion molecule, EpCAM）表面抗原的CTC，而CTC会出现丢失EpCAM表面抗原的现象；②不同恶性肿瘤携带EpCAM抗原的CTC比例不同，部分癌症亚型的CTC细胞表面甚至不表达EpCAM抗原。其他的CTC分离方法如阴性富集法和ISET方法目前也较为常用，这两种方法不基于特定的分子标志物，因而CTC检出率相对较高，但仅仅能对CTC进行计数，无法进一步对CTC进行分型，因而对临床上进行转移复发研究帮助非常有限^[9, 10]^。本研究选用CanPatrol技术对CTC进行筛选，该技术利用纳米滤膜及RNA原位杂交方法，能够在对不同类型的CTC进行鉴别同时，对CTC表面不同基因的表达进行检测。

细胞的上皮-间质转化（epithelial-mesenchymal transition, EMT）指的是通过调控细胞表面粘附分子的表达，使细胞更具有侵袭性及迁徙性，这一过程的经典理论由Hay^[11]^在1995年提出。经过上述过程后CTC可分为上皮型、间质型及混合型三种类型，并且肿瘤细胞可获得突破基底膜进入系统循环的能力，但只有一小部分肿瘤细胞可最终通过重新转变为上皮表型（mesenchymal to epithelial transition, MET）转化为远处器官的转移灶。CTC的EMT过程在肿瘤的转移过程中起着非常重要的作用。目前已经证实EMT与肿瘤侵袭性、化疗药物耐药以及较差的临床预后相关^[12]^。

在对所有数据与CTC相关性进行的秩相关检验中，我们发现CTC阳性率与患者的吸烟史相关（*Spearman*系数=0.434，*P*=0.016）。根据*Spearman*系数可以看出，既往有吸烟史的患者，血液中CTC阳性率较高，且具有统计学差异。我们并未在既往文献报道中检索到与上述结果一致的结论^[13]^。从表 1中不难看出，吸烟的患者均为男性，且秩相关检验证实吸烟与肺鳞癌相关（*Spearman*系数=0.473，*P*=0.008），这与我们既往所熟知的NSCLC的临床特点及我国吸烟人群主要以男性为主一致。既往有研究认为CTC数量与NSCLC的病理类型无关^[14, 15]^。此次秩相关分析的结果中我们也并未发现外周血CTC的阳性率与性别和肿瘤类型相关（*P*=0.384, *P*=0.164），同时，也并未发现外周血CTC数量与性别和肿瘤类型相关（*P*=0.224, *P*=0.104）。

在目前的分析结果中发现，吸烟的患者肺部肿瘤的直径较大（*Spearman*系数=0.412，*P*=0.024），在将肿瘤直径的数据类型由二分类变量转变为连续变量再次进行秩相关检验后，所得到的结果也证实了上述的结果（*Spearman*系数=0.396，*P*=0.030）。结合不同类型NSCLC的临床特点进行分析，吸烟与肺鳞癌的发生密切相关，肺鳞癌以中央型为主，且往往因出现如咯血、阻塞性肺炎等临床症状时就诊，此时肿瘤直径较大，往往超过3 cm。以往研究认为外周血中CTC的数量与肿瘤的分期相关^[13, 15]^。相较于早期患者，中晚期的NSCLC患者外周血中更容易检测出CTC，且数量更多。但是此次研究中，我们并未发现CTC阳性率与NSCLC分期之间存在相关性（*Spearman*系数=0.084，*P*=0.660）。在将淋巴结转移数量与其他变量进行秩相关检验时我们发现其与性别相关（*Spearman*系数=0.371，*P*=0.044），即男性患者淋巴结转移数量更多且具有统计学差异。Decaluwe等^[16]^的研究发现，即使是临床分期考虑为Ⅰ期的NSCLC患者，中央型肺癌的淋巴结转移的转移率更高，更容易出现淋巴结转移。结合本组患者的临床特点分析，本组男性患者中以中央型肺鳞癌为主，因此出现淋巴结转移的几率更大。

FGFR1属于跨膜酪氨酸激酶受体（receptor tyrosine kinase, RTK），由胞外区、跨膜螺旋区及具有酪氨酸激酶活性的胞内区组成。作为庞大复杂分子信号系统的一部分，参与细胞分化、生长、组织发生发展、血管生成及伤口愈合等多种过程中^[7, 8]^，且大部分成纤维生长因子（fibroblast growth factor, FGF）通过自分泌或旁分泌的形式发挥其生理作用。一项包括3, 131份肿瘤标本及26个癌种的研究发现，FGFR1扩增率为10%^[17]^。FGF与FGFR结合使其磷酸化，进而激活包括PI3K、ERK/MAPK及JNK等在内的信号通路来产生一系列生物学效应^[10, 18, 19]^，且上述通路是多种肿瘤生长、转移及血管生成的中心环节。研究认为FGF2、FGF9、FGFR1 Ⅲc及FGFR2 Ⅲc在NSCLC的发生发展过程中具有一定的作用^[7, 20-22]^。由此可见，越来越多的证据表明FGF家族与FGFR组成的自分泌和旁分泌系统参与到NSCLC发生发展的各个环节中，并有着重要的作用。

FGFR1在肺鳞癌中FGFR1扩增的发生率在10.7%-20.7%之间^[7, 8, 23-25]^，在肺腺癌中发生率较低，大约在3%左右^[8, 25, 26]^。一项纳入了329例Ⅰ期-Ⅱ期的淋巴结阴性的NSCLC研究表明，肺鳞癌中FGFR1扩增率达20.7%且具有统计学意义^[8]^，且大多数研究表明FGFR1扩增与NSCLC亚型相关且多发生于肺鳞癌亚型的患者^[8, 17, 26, 27]^。多数研究^[8, 17, 26-29]^发现FGFR1扩增状态更多见于男性患者，且与吸烟史相关。也有研究^[28]^发现在不同NSCLC亚型的患者中，FGFR1的扩增情况与吸烟史无关。Cihoric等^[8]^的数据表明随着肿瘤直径、T分期以及肿瘤-淋巴结-转移（tumor-node-metastasis, TNM）分期的升高，FGFR1的突变率也在升高，且后者与前三者之间的这一关系具有统计学意义。

目前发现不同类型的CTC在出现远处转移患者全身治疗过程中的药物敏感性也不同，因此认为CTC的分子特点可为肿瘤转移以及抗肿瘤药物筛选提供新的思路^[30]^。本组患者利用CanPatrol技术，在不同类型CTC上检测FGFR1的表达程度。根据不同的表达程度，将FGFR1分为无表达、低表达、中表达及高表达。在进行统计学分析时我们定义FGFR1低、中、高表达状态为基因表达状态。秩相关检验中并未发现*FGFR1*基因表达状态与本组患者包括肿瘤类型、肿瘤分期、吸烟史等在内的临床特点相关。利用*Mann-Whitney U*检验中也并未发现不同病理类型的肺癌中FGFR1的表达存在着差异（*P*=0.806）。我们认为这主要与肿瘤异质性相关，即在原发肿瘤病灶脱落的CTC细胞上，FGFR1的表达与原发灶之间存在着差异。我们曾假设在CTC发生EMT后*FGFR1*基因的表达会相应增高，或CTC阳性率及数量较高的患者FGFR1的表达会相应增加，但在秩相关检验及*Kruskal-Wallis*检验中，我们并未发现不同类型CTC细胞中FGFR1的表达存在差异（*P*=0.202, *P*=0.094）。

综上所述，本研究共纳入30例NSCLC患者，在对这30例患者外周血进行CTC检测及分型的基础上，我们检测了每位患者CTC上FGFR1表达的情况。利用秩相关分析发现外周血CTC的数量与吸烟史相关，既往有吸烟史的患者外周血CTC数量相应增加，但在进一步的分析中并未发现不同病理类型中外周血CTC的数量以及CTC上*FGFR1*基因表达上有何区别。FGFR1的表达水平与外周血CTC数量并无相关性，且在不同类型的CTC上FGFR1的表达情况也并没有具有统计学意义的差别。本研究受限于较小的样本量，我们期待着更大样本量及纳入随访数据后可得出与CTC及*FGFR1*基因表达相关的更多具有临床应用意义的结论。
